# Cocoon Syndrome: A Clinical Case of Intestinal Obstruction Highlighting the Diagnostic and Therapeutic Importance of Exploratory Laparoscopy in the Face of Ambiguous Imaging

**DOI:** 10.7759/cureus.114495

**Published:** 2026-08-13

**Authors:** Amanda I Wosiack Menin, Ricardo Padilla, Gabriela Proaño, Sofía Pinedo, Luana Wosiack

**Affiliations:** 1 Surgery, Hospital El Carmen Dr. Luis Valentín Ferrada, Santiago, CHL; 2 Medicine, Universidad Finis Terrae, Santiago, CHL; 3 General Surgery, Universidad Diego Portales, Santiago, CHL; 4 General Surgery, Universidad Finis Terrae, Santiago, CHL; 5 General Medicine, Universidad de Santiago de Chile, Santiago, CHL

**Keywords:** abdominal adhesions, ambiguous imaging, intestinal obstruction, laparoscopy, sclerosing peritonitis

## Abstract

Cocoon syndrome, also known as sclerosing encapsulating peritonitis (SEP), is a rare condition characterized by the encapsulation of small bowel loops by a thick fibrocollagenous membrane. Its non-specific clinical presentation and ambiguous findings on conventional imaging often lead to significant diagnostic delays or misdiagnosis, frequently being mistaken for internal hernias or neoplastic processes. We present the case of a 61-year-old male patient with no prior abdominal surgical history who presented with a six-month history of recurrent abdominal pain, vomiting, and weight loss. While magnetic resonance imaging and computed tomography suggested a high-grade intestinal obstruction, they could not definitively rule out an underlying malignancy. Exploratory laparoscopy was performed, revealing a dense fibrin mantle encapsulating the small bowel loops and a carnified greater omentum. Extensive laparoscopic blunt and bipolar adhesiolysis was successfully carried out, achieving complete intestinal release. The patient experienced an excellent postoperative recovery and was discharged five days after the procedure. This case highlights the pivotal role of exploratory laparoscopy as a minimally invasive tool that provides both definitive diagnostic confirmation and effective surgical treatment, reducing the morbidity associated with traditional laparotomy in cases of ambiguous intestinal obstruction.

## Introduction

Cocoon syndrome, or sclerosing encapsulating peritonitis (SEP), is an uncommon condition that typically manifests as recurrent acute, subacute, or chronic episodes of intestinal obstruction [1-3]. Initially described by Owtschinnikow in 1907 [4], the term "abdominal cocoon" was later coined by Foo et al. to describe a primary or idiopathic form of the disease [5]. The name refers to the characteristic appearance of a thick fibrocollagenous membrane that envelops the small intestine and, occasionally, other intra-abdominal organs [6,7].

The etiology of SEP is multifactorial and divided into two entities: primary (idiopathic), which is extremely rare and of unknown cause, and secondary, which is associated with conditions such as peritoneal dialysis or abdominal tuberculosis, with the latter being the most common [3]. The syndrome most frequently affects men between the ages of 20 and 40 [5]. Based on the extent of the membrane, it is classified into Type I (partial involvement of the small bowel), Type II (total involvement), and Type III (total small bowel involvement plus other organs such as the appendix, cecum, or ovaries) [3,6,7]. While this classification aids in description, it does not strictly dictate medical management [8].

The exact pathophysiology remains unknown, though the role of cytokines and fibroblasts in peritoneal fibrosis and neoangiogenesis is well-documented [1,9]. Some theories suggest that a dysplastic greater omentum, present in nearly all cases, may result from congenital omental dysplasia, leading to a membrane that adheres to the intestines and causes dysfunction [8]. Clinical manifestations are non-specific and include abdominal pain, anorexia, vomiting, constipation, and weight loss.

Preoperative diagnosis is notoriously difficult, almost impossible, and rarely achieved before surgery [7,9]. While most cases remain asymptomatic until an obstructive event occurs, modern imaging has improved preoperative detection [3,10]. Contrast-enhanced computed tomography (CT) remains the gold standard, often showing dilated bowel loops within a thick peritoneal membrane (>2 mm), adhesions, and ascites [10,11]. Laparoscopy plays a crucial role, serving as both a diagnostic and therapeutic tool in cases of clinical uncertainty [8]. While mild symptoms may be managed conservatively, surgery, traditionally an exploratory laparotomy with membrane excision and adhesiolysis, is required for refractory cases [2,3,7]. However, minimally invasive approaches are increasingly preferred to reduce complications like fistulas or sepsis [2].

## Case presentation

A 61-year-old male patient with a medical history of arterial hypertension and coronary heart disease, and notably no history of prior abdominal surgeries, presented to an outpatient clinic with a six-month history of diffuse, intermittent abdominal pain. The pain had intensified in recent weeks, accompanied by vomiting and unquantified weight loss.

**Figure 1:**
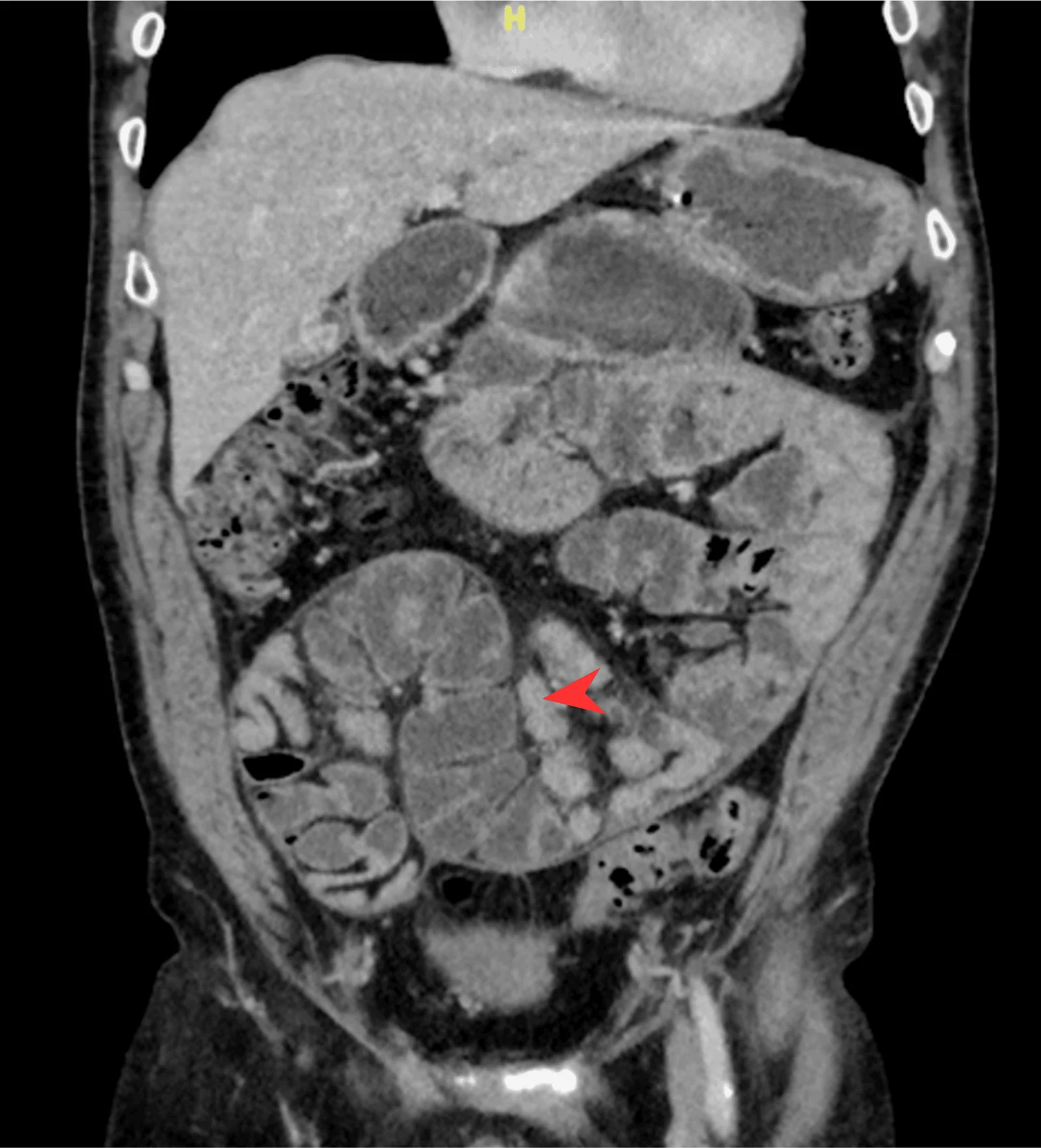
Contrast-enhanced CT of the abdomen and pelvis, coronal view. Significant dilation of multiple small bowel loops is observed, indicative of high-grade intestinal sub-occlusion primarily localized in the right upper quadrant. Additionally, a notable clustered distribution of bowel loops is evident within the central abdomen and the right iliac fossa, indicated by a red arrow.

Upon referral to the emergency department, the patient was hemodynamically stable, afebrile, and nauseated. Physical examination revealed a thin-appearing man in good general condition. The abdomen was soft, depressible, and non-tender, with active bowel sounds and no palpable masses or lymphadenopathy. An outpatient magnetic resonance imaging (MRI) report indicated signs of high-grade intestinal obstruction in the proximal jejunum, raising suspicion for a possible intrinsic neoplastic lesion. A follow-up CT scan showed signs of intestinal sub-occlusion suggestive of a transmesenteric internal hernia (Figure 1). Laboratory results were within normal ranges.

**Figure 2:**
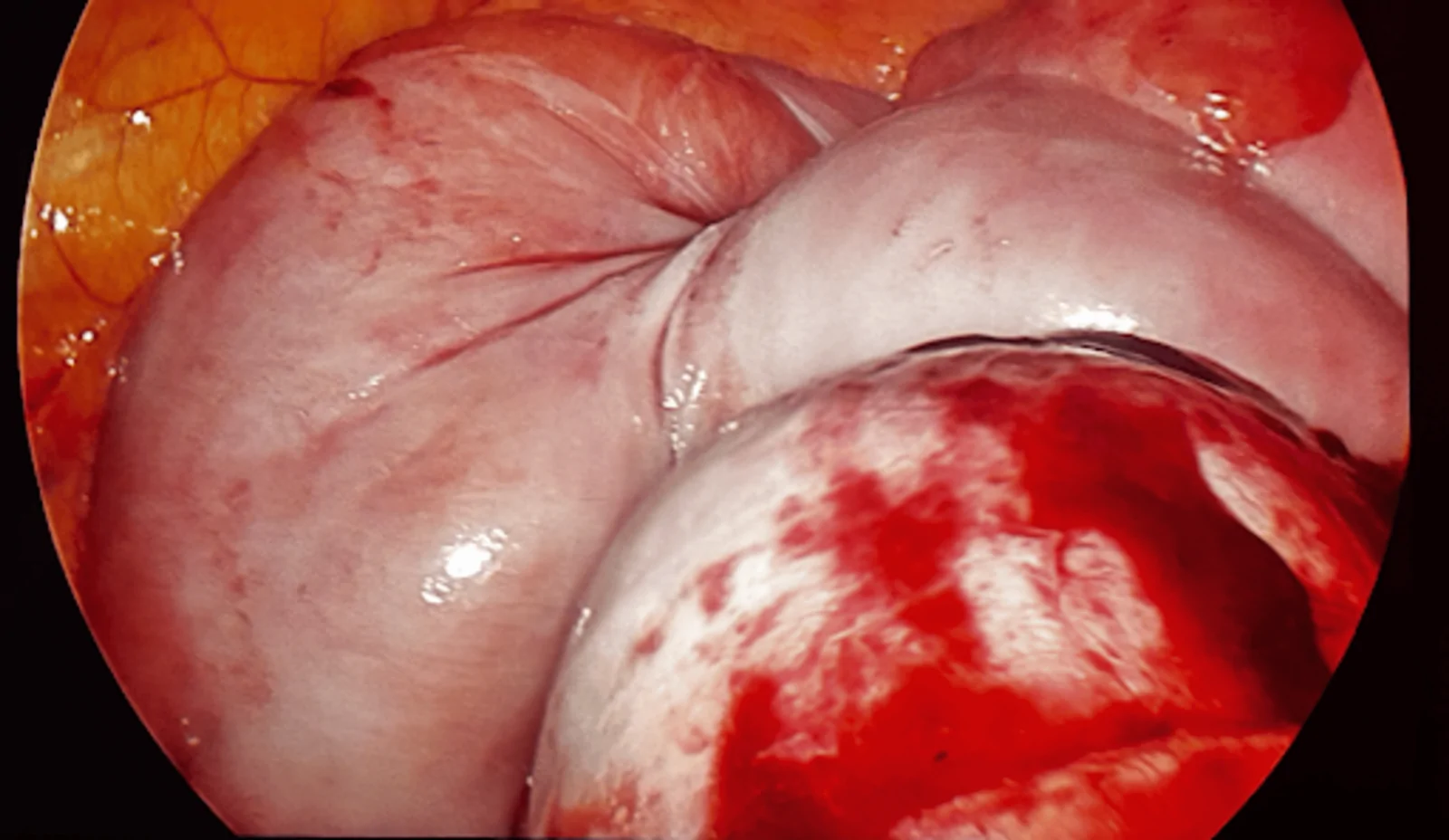
Intraoperative laparoscopic view A thick, opaque, and characteristic whitish-grey fibrocollagenous membrane (capsule) is seen encasing numerous matted small bowel loops. The bowel is clustered into a distinct 'cocoon' pattern, covered by a tough, leathery pale sheath that obscures the underlying detailed anatomy.

The patient was hospitalized for surgical management. Exploratory laparoscopy revealed a large, dense fibrin mantle forming inter-loop adhesions that created at least three distinct "cocoons" in the right iliac fossa and left hypochondrium, classifying this as a Type I Cocoon syndrome due to the partial encapsulation (Figure 2). 

**Figure 3:**
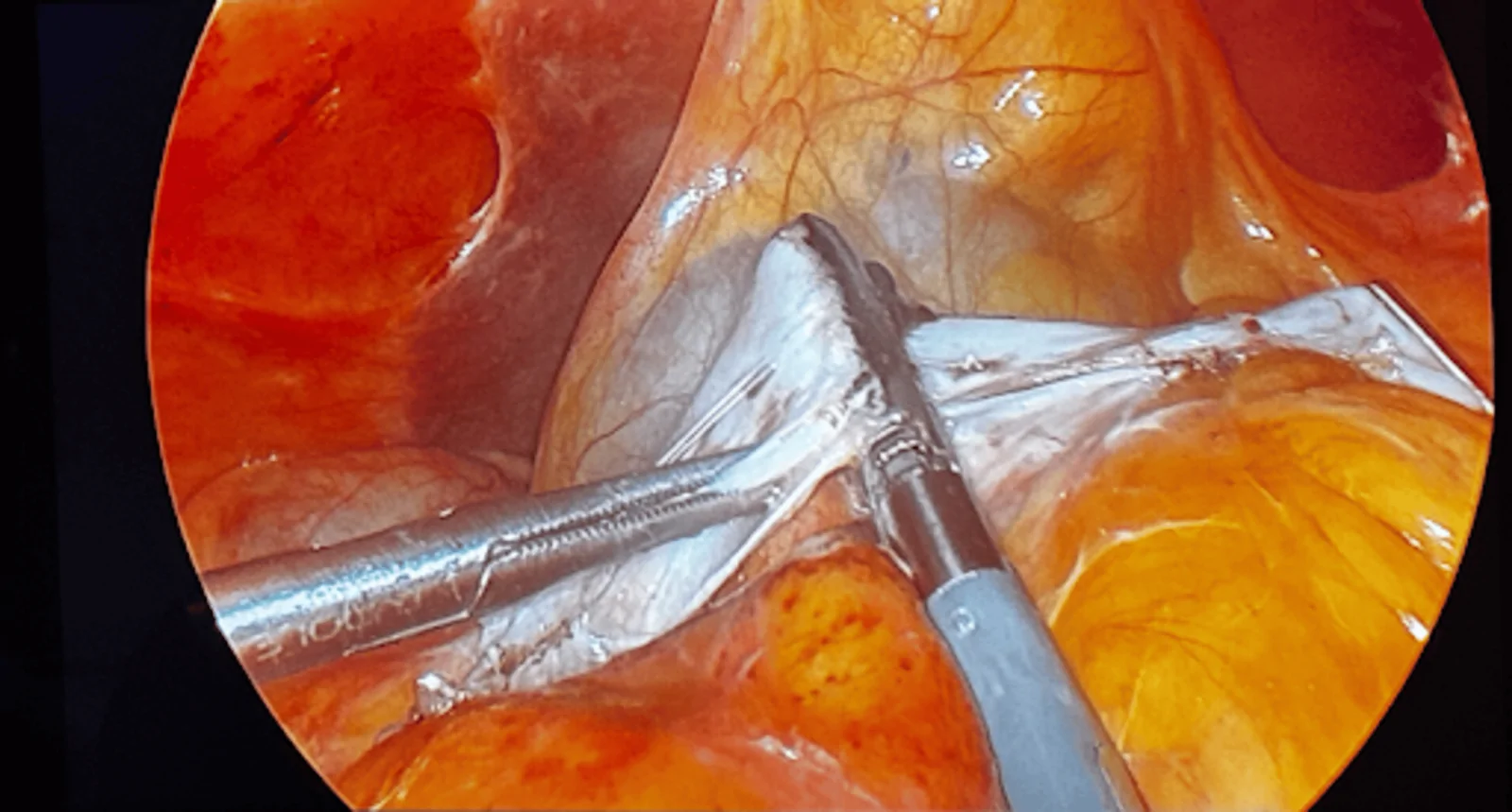
Intraoperative laparoscopic view showing the meticulous resection of the fibrocollagenous membrane. A grasper provides counter-traction as a bipolar energy device is used to release the small bowel loops from the encapsulating 'cocoon' sheath.

Dilation of the small bowel loops was evident, while the colon was collapsed and the greater omentum appeared carnified. Extensive adhesiolysis was performed using both blunt dissection and a bipolar device (Figure 3), successfully releasing the dysplastic omentum and the encapsulated bowel loops. Peristalsis was confirmed, along with the passage of intestinal content into the colon. The patient recovered favorably, with immediate resolution of nausea and progressive reduction of pain. Normal intestinal transit was restored, and he was discharged on the fifth postoperative day without complications.

## Discussion

Abdominal cocoon syndrome, or SEP, remains a formidable diagnostic and therapeutic challenge primarily due to its insidious, non-specific presentation and relative scarcity in daily clinical practice [1,3,7]. While primary or idiopathic SEP typically manifests in younger men between the second and fourth decades of life [7], our case illustrates an atypical presentation in a 61-year-old man with no predisposing factors or prior surgical history. The pathophysiology of idiopathic SEP is not fully elucidated; however, leading hypotheses suggest congenital omental dysplasia or inflammatory cytokine-mediated peritoneal fibrosis, which eventually leads to the formation of a dense fibrocollagenous sheath encasing the small intestine [1,8,11].

Preoperative diagnosis of SEP is notoriously difficult, with most cases identified incidentally during emergency exploratory procedures [3,7,8]. Although cross-sectional imaging has advanced, distinguishing SEP from other causes of small bowel obstruction, such as internal hernias, peritoneal carcinomatosis, or primary gastrointestinal malignancies, remains troublesome [2,9,10]. CT is considered the gold standard imaging modality, classically demonstrating fluid-filled, dilated small bowel loops concentrated in the center of the abdomen within a thick membrane (>2 mm) [9,10]. In our patient, initial MRI suggested a proximal jejunal obstruction possibly secondary to an intrinsic neoplastic lesion, whereas subsequent contrast-enhanced CT raised suspicion for a transmesenteric internal hernia. This diagnostic ambiguity underlines the limitations of imaging alone in differentiating encapsulated fibrous sheaths from mesenteric defects or mass lesions [2,9].

Historically, the standard surgical intervention for symptomatic or refractory SEP consisted of open exploratory laparotomy involving complete membrane excision and extensive enterolysis [2,3,7]. However, traditional open approaches carry a substantial burden of postoperative complications, including prolonged ileus, enterocutaneous fistulas, bowel perforation, and recurrent adhesion formation [2]. In recent years, minimally invasive techniques have gained traction as safe alternatives in selected cases [2,11]. In our patient, exploratory laparoscopy allowed for immediate, high-definition direct visualization of the characteristic whitish-grey fibrocollagenous membrane and distinct "cocoons" encasing the jejunum and ileum. Furthermore, utilizing a combination of laparoscopic blunt dissection and bipolar electrocautery enabled complete lysis of the thick sheath and release of the carnified greater omentum without intraoperative bowel injury.

Compared to open laparotomy cases reported in the literature, which often necessitate extended hospital stays and carry higher morbidity rates [3,7,8], the laparoscopic approach in our case facilitated rapid functional recovery, immediate resolution of obstructive symptoms, early restoration of intestinal transit, and discharge on postoperative day five without adverse events. Therefore, this case highlights that even in complex presentations of chronic bowel obstruction with misleading imaging, exploratory laparoscopy serves a crucial dual function as both a definitive diagnostic modality and an effective, low-morbidity therapeutic strategy [2,11].

## Conclusions

Abdominal cocoon syndrome remains a significant surgical trap, primarily due to its rarity and the absence of pathognomonic clinical features. As demonstrated in this case, the lack of traditional risk factors, such as prior abdominal surgeries or peritoneal dialysis, should not lead the clinician to rule out this entity. When advanced imaging like CT or MRI proves inconclusive or raises suspicion of complex internal hernias or malignancy, exploratory laparoscopy emerges as the definitive diagnostic and therapeutic bridge. It provides the surgeon with the necessary visualization to navigate the dense fibrous landscape of the encapsulated bowel while offering a minimally invasive path to full recovery. This case serves as a reminder that clinical persistence and the early adoption of a laparoscopic approach are essential to untangling the diagnostic uncertainty of chronic intestinal obstruction and preventing the morbidity of an unnecessary or delayed laparotomy.
